# Supplementary material for: Bone marrow adiposity modulation after long duration spaceflight in astronauts
Source: Nat Commun. 2023 Aug 9;14:4799. doi: 10.1038/s41467-023-40572-8 (PMC10412640; doi:10.1038/s41467-023-40572-8)
Supplement: Supplementary file 1 — Supplementary Information [file 41467_2023_40572_MOESM1_ESM.pdf]

Bone Marrow Adiposity Modulation After Long Duration Spaceflight in Astronauts

Supplementary Information

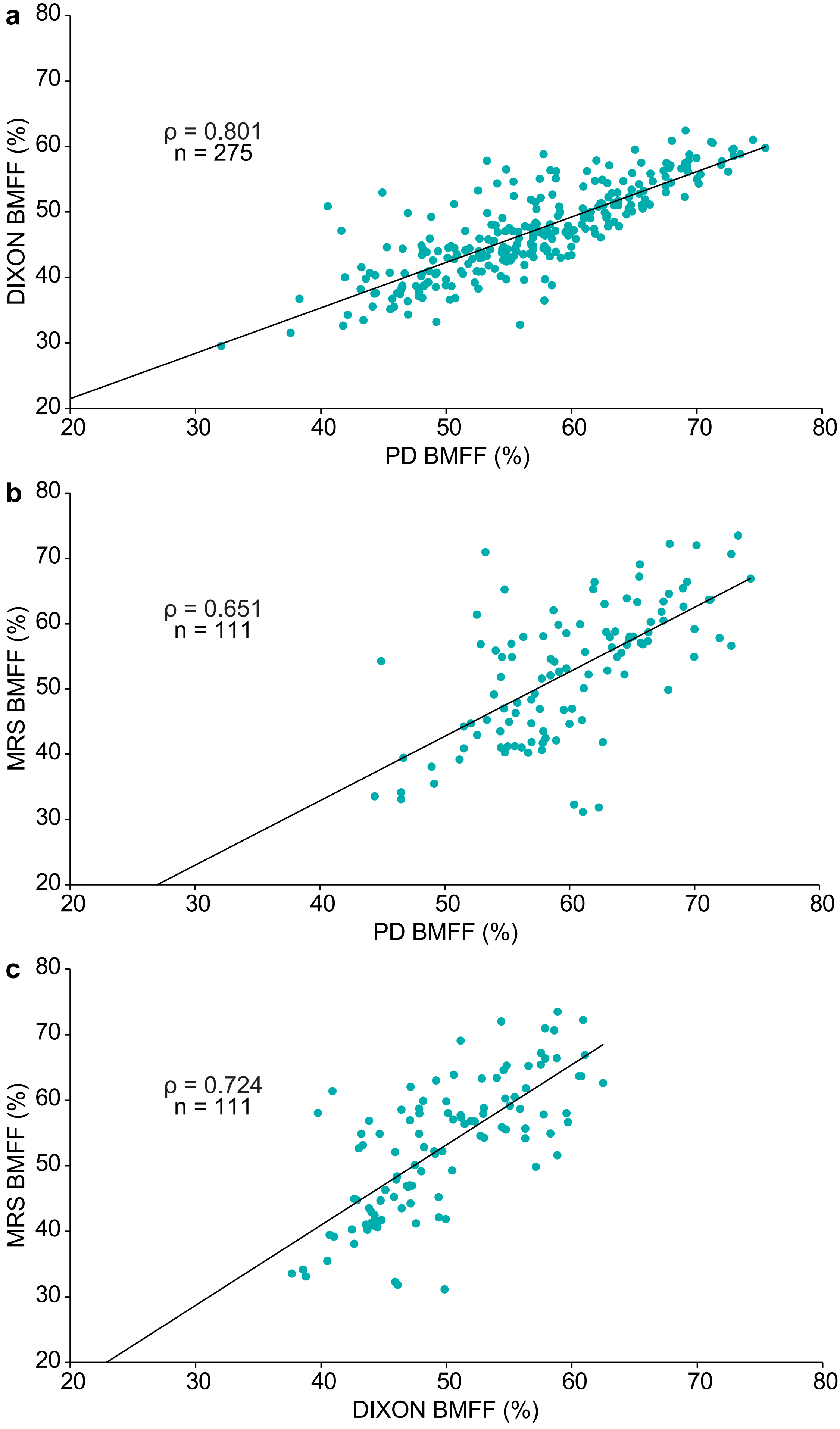

Supplementary Figure 1. Correlation between 3 MR quantitative techniques for BMFF. The 3 quantitative MR techniques used to quantify lumbar BMFF were strongly correlated (Spearman's correlation coefficient; ρ). **a.** PD-DIXON (ρ=0.801), **b.** PD-MRS (ρ=0.651), **c.** DIXON-MRS (ρ=0.724). PD and DIXON included 5 lumbar vertebrae (L1, L2, L3, L4, and L5) whereas MRS were obtained from L4 and L5.

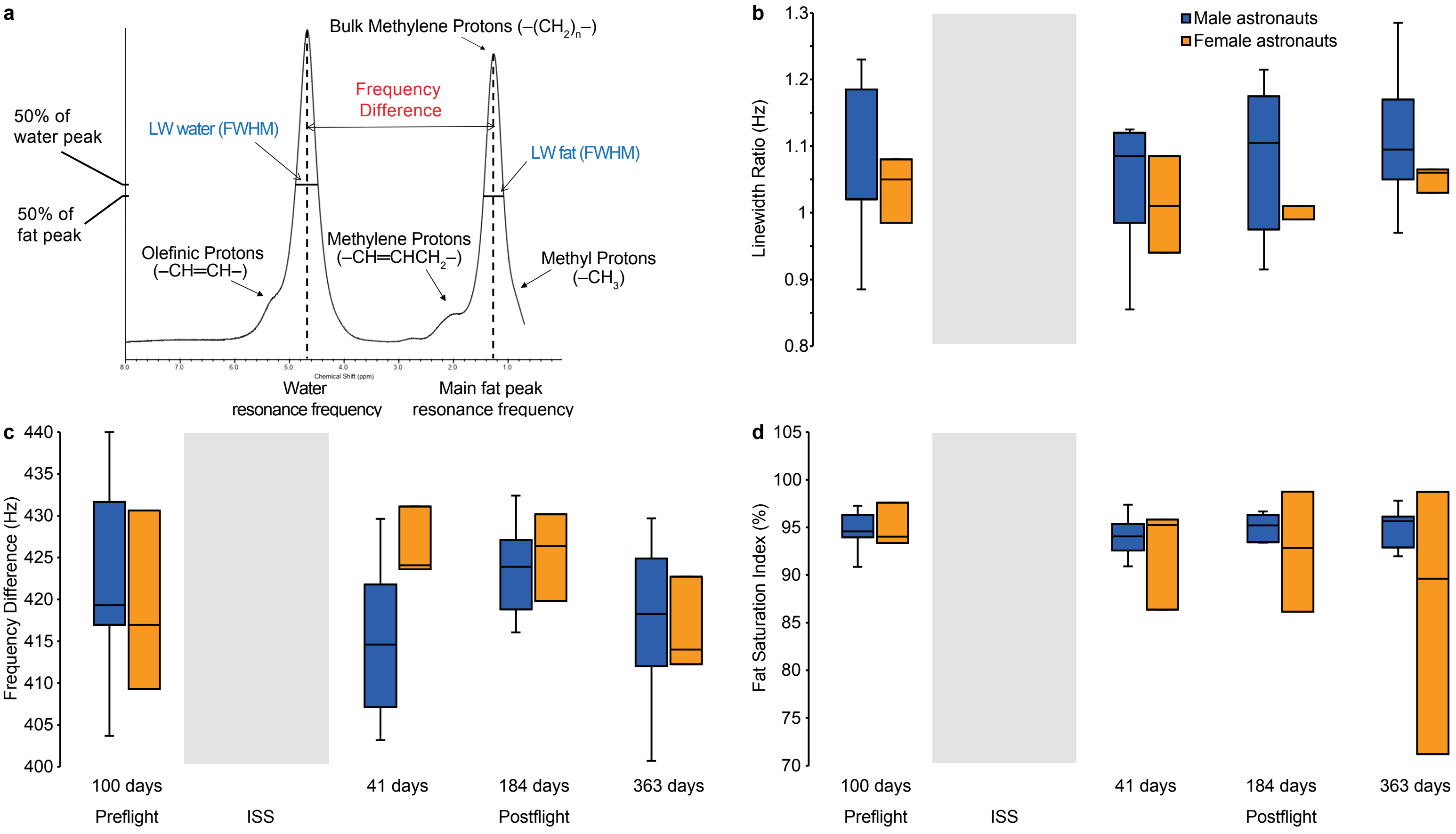

Supplementary Figure 2. Linewidth ratio (LWR), frequency differences and fat saturation from MR spectra before spaceflight and 41 days, 184, and 363 days postflight in 14 astronauts. a. Representation of a proton magnetic resonance spectrum of L4 with the water peak (left) at 4.7 ppm and the main fat peak (right) at 1.3 ppm. An increase in LWR can reveal a decrease in extracellular water and/or increase of iron in the bone marrow compared to baseline. A decrease in frequency difference can reveal a decrease in iron content in the bone marrow compared to baseline. b. Linewidth ratio; c. Frequency differences. Both LWR and frequency differences remained unchanged in both male and female astronauts compared to baseline with no gender-based differences at all experimental time points (all P>0.05). The stable water signal allowed attributing the change in fat fraction to changes in marrow adipose tissue. d. Fat saturation index. Mean L4 and L5 fat saturation index measured using MR spectroscopy. The fat saturation index did not change significantly 41, 184, and 363 days after an average 167-day space missions compared to preflight data (all P>0.05). Therefore, the BMA modulation was not attributable to a change in fatty acid saturation of regulated BMAT. LW: linewidth, FWHM: full width at half maximum. Shaded grey areas correspond to time onboard the International Space Station. Boxes show 2 quartiles around the median. Whiskers indicate minimum and maximum values excluding outliers.

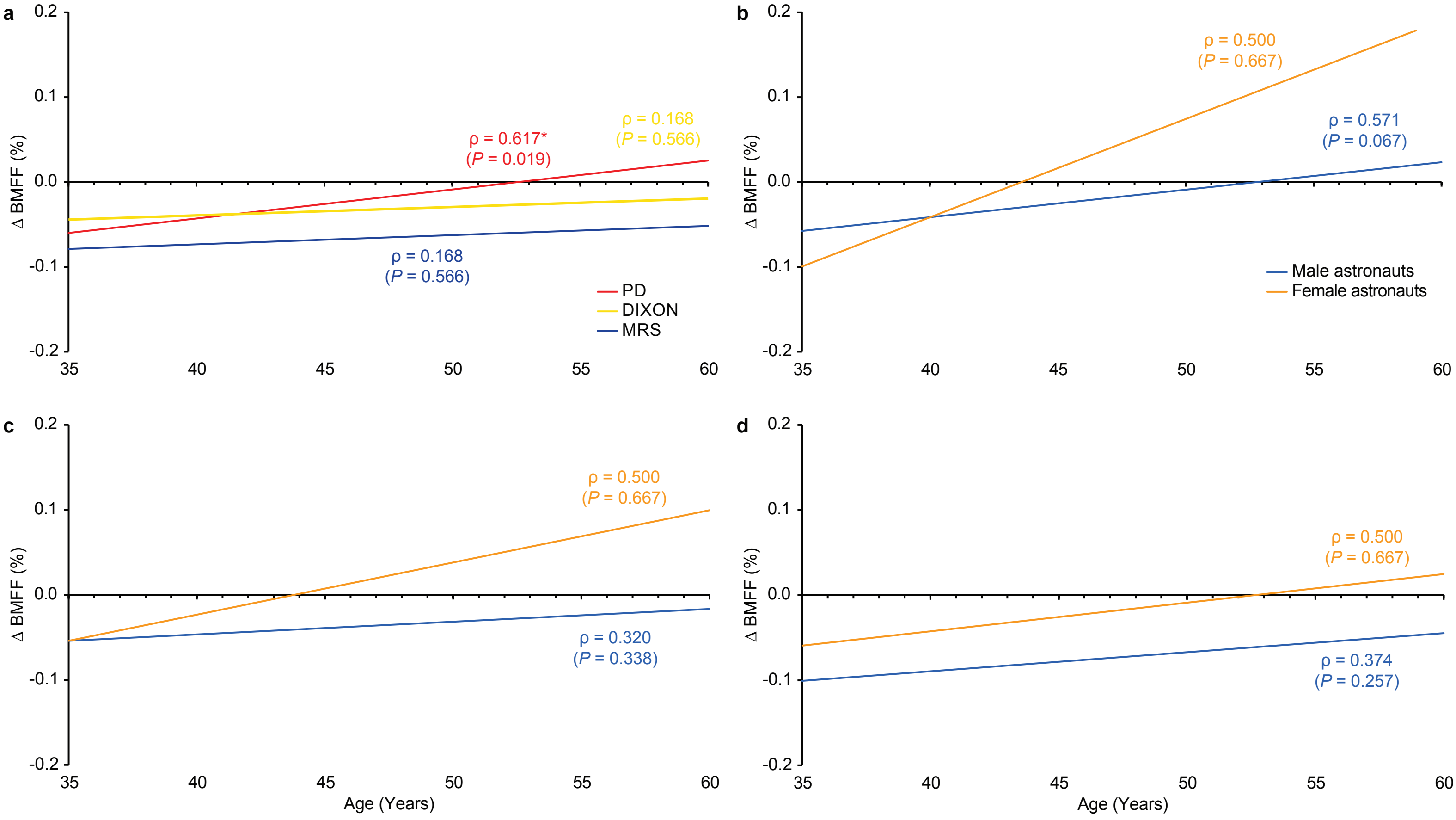

Supplementary Figure 3. Effect of astronaut age on the change in lumbar vertebrae BMFF at 41 days postflight. **a.** Spearman's correlation of the astronaut cohort using average data from 3 different MR techniques: proton density (PD) with and without fat saturation, DIXON, and spectroscopy (MRS); **b.** Sex specific Spearman's correlations using PD; **c.** Sex-specific Spearman's correlations using DIXON; and **d.** Sex-specific Spearman's correlations using MRS. Age was moderately correlated with change in BMFF in all astronauts as measured using PD. **P*<0.05 between age and change in lumbar BMFF by Spearman's correlation.

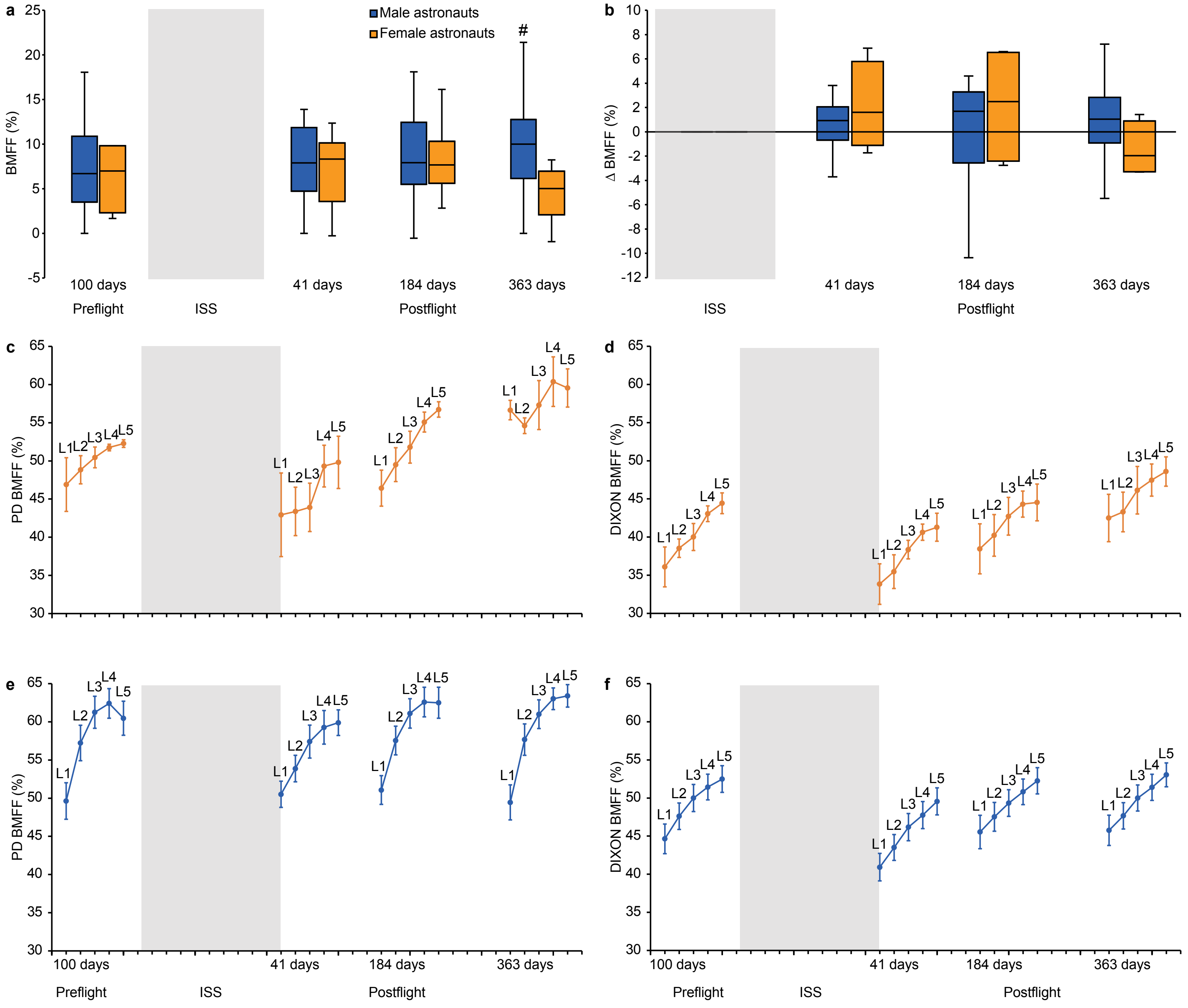

Supplementary Figure 4. BMA gradient preflight and 41, 184, and 363 days postflight after 167 days mission on the ISS. **a.** Average vertebral BMA gradient in male and female astronauts. **b.** Change in BMA gradient from preflight measures. Long-duration space missions did not flatten the gradient in lumbar vertebrae BMA. **c-d:** Lumbar BMA gradient in female astronauts with **c.** PD and **d.** DIXON. **e-f:** Lumbar BMA gradient in male astronauts with **e.** PD and **f.** DIXON. Long-duration space flight modulated BMA, but the L5-L1 BMA gradient showed a high degree of preservation. Shaded areas correspond to time onboard the International Space Station. Boxes show 2 quartiles around the median. Whiskers indicate minimum and maximum values excluding outliers. #*P*<0.05 between male and female astronauts by two-sided Mann-Whitney U test (n=14) with no adjustment for multiple comparisons.

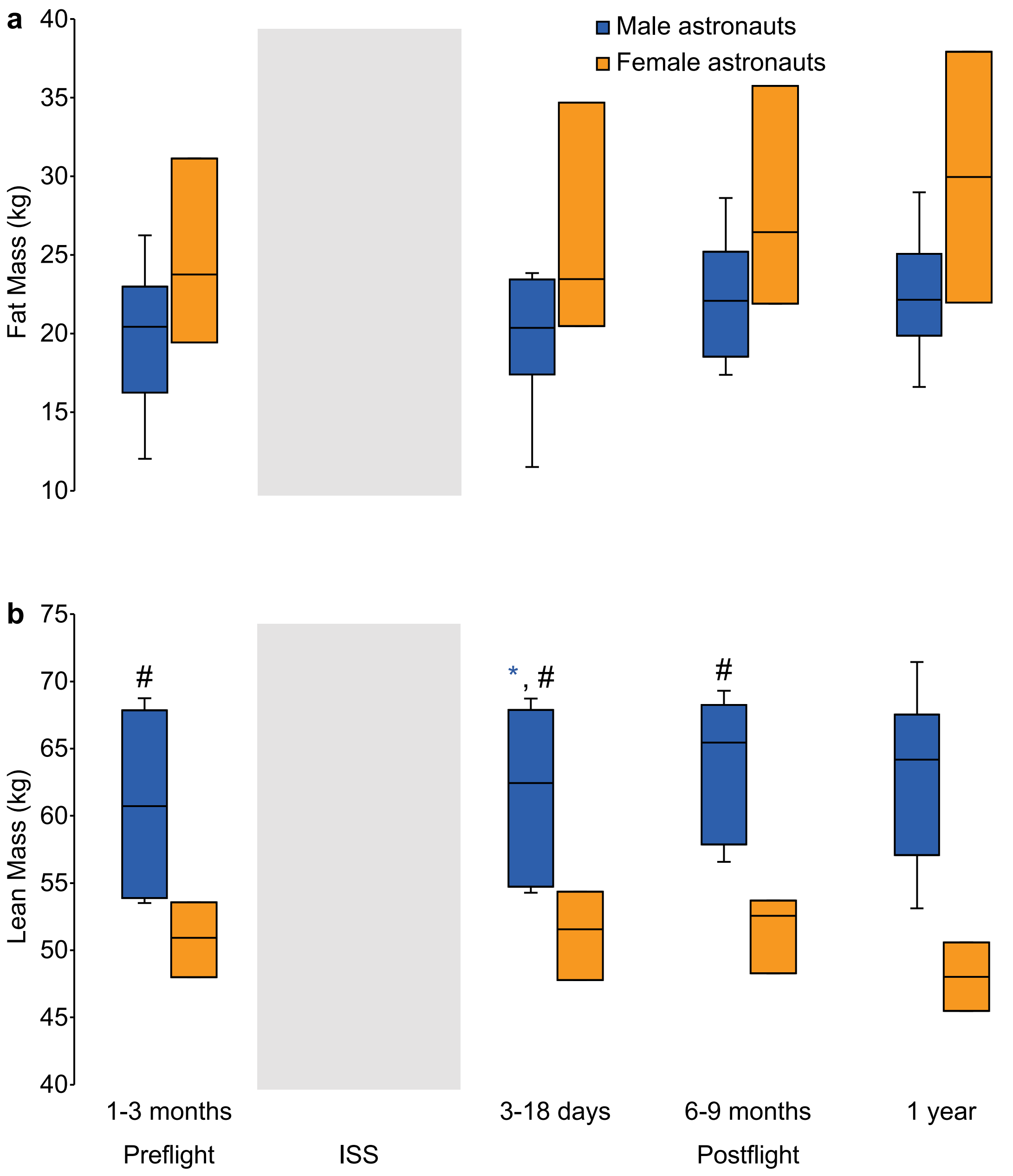

Supplementary Figure 5. Correlation between BAM and peripheral fat. Long-duration space mission modulation of BMA showed no systemic or paradoxical relationship with whole body fat mass. **a.** Whole body fat mass; and **b.** Whole body lean mass. Lean mass in male astronauts 3-18 days postflight were statistically higher than preflight (*P*<0.05). Both fat and lean masses were unchanged compared to preflight at all experimental time points in female astronauts. Shaded area corresponds to time onboard the International Space Station. Boxes show 2 quartiles around the median. Whiskers indicate minimum and maximum values excluding outliers. **P*<0.05 compared to preflight values by two-sided Wilcoxon Signed Rank test (n=14) with no adjustment for multiple comparisons. #*P*<0.05 between male and female astronauts by two-sided Mann-Whitney U test (n=14) with no adjustment for multiple comparisons.

Supplementary Tables

Supplementary Table 1. Returning from prolonged spaceflight modulates bone marrow adiposity at the lumbar spine in 14 astronauts measured using 3 quantitative magnetic resonance techniques. MAT in percentage point of the vertebral marrow (SD).

	Technique	Preflight -100±43 days	Postflight 41±6 days	Postflight 184±15 days	Postflight 363±25 days
All astronauts (n=14)	PD	56.7 (6.6)	54.2 (7.2)	57.6 (6.0)	58.8 (4.8)
	DIXON	47.6 (6.4)	44.1 (6.3)*	47.5 (6.3)	48.6 (5.5)
	MRS	54.8 (10.1)	47.8 (8.7)*	53.4 (9.4)	55.1 (9.9)
	All techniques	53.0 (7.2)	48.7 (6.5)*	52.8 (6.3)	54.2 (6.0)
Male astronauts (n=11)	PD	58.4 (6.3)	56.5 (5.9)#	59.1 (5.8)	59.1 (5.2)
	DIXON	49.5 (5.9)#	45.8 (5.9)*#	49.0 (6.0)	49.5 (5.7)
	MRS	58.3 (8.3)#	50.6 (7.5)*#	53.9 (10.2)	55.5 (10.0)
	All techniques	55.4 (6.2)#	50.6 (5.5)*#	54.0 (6.7)	54.7 (6.1)
Female astronauts (n=3)	PD	50.2 (1.6)	45.9 (5.8)#	51.9 (2.4)	57.7 (3.3)
	DIXON	40.8 (2.2)#	37.9 (3.0)#	42.1 (4.3)	45.6 (4.2)
	MRS	41.9 (2.4)#	37.6 (3.8)#	51.6 (7.0)	53.5 (11.7)
	All techniques	44.3 (1.9)#	41.8 (5.4)*#	48.5 (0.4)*	52.3 (6.2)*

PD: Proton density with and without fat saturation; MRS: MR spectroscopy. Average (SD). *$P<0.05$ compared to preflight bone marrow adiposity by two-sided Wilcoxon Signed Rank test (n=14) with no adjustment for multiple comparisons. #$P<0.05$ between male and female astronauts by two-sided Mann-Whitney U test (n=14) with no adjustment for multiple comparisons.

Supplementary Table 2. Bone mineral density, T-score, and Z-score in 14 astronauts using dual energy x-ray absorptiometry (DXA).

	Measures	Preflight 1-3 months	Postflight 3-18 days	Postflight 6-9 months	Postflight 1 year
All astronauts (n=14)	BMD (g/cm^2)	1.07 (0.11)	1.03 (0.12)*	1.07 (0.12)#	1.04 (0.10)
	T-score	-0.12 (1.14)#	-0.42 (1.20)*	-0.09 (1.24)#	-0.32 (1.02)
	Z-score	0.15 (1.16)	-0.15 (1.20)*#	0.23 (1.25)#	0.01 (1.06)
Male astronauts (n=11)	BMD (g/cm^2)	1.04 (0.09)	1.00 (0.10)*	1.02 (0.10)*	1.02 (0.11)
	T-score	-0.51 (0.86)	-0.81 (0.94)*	-0.68 (0.85)	-0.61 (0.94)
	Z-score	-0.22 (0.94)	-0.52 (0.98)	-0.35 (0.93)	-0.27 (1.02)
Female astronauts (n=3)	BMD (g/cm^2)	1.17 (0.12)	1.14 (0.14)	1.18 (0.12)	1.12 (0.06)
	T-score	1.17 (1.12)	0.87 (1.20)	1.11 (1.05)	0.70 (0.57)
	Z-score	1.36 (1.10)	1.10 (1.15)	1.40 (1.01)	1.00 (0.42)

Average (SD). *P<0.05 compared to preflight by two-sided Wilcoxon Signed Rank test (n=14) with no adjustment for multiple comparisons. #P<0.05 between male and female astronauts by two-sided Mann-Whitney U test (n=14) with no adjustment for multiple comparisons.

Supplementary Table 3. Pearson's correlation coefficient and Spearman's rho between change in marrow adipose tissue and age 41 days postflight.

		Pearson's correlation coefficient	Spearman's rho
PD	All astronauts (n=14)	0.463	0.617*
	Male astronauts (n=11)	0.429	0.571
	Female astronauts (n=3)	0.539	0.500
DIXON	All astronauts (n=14)	0.226	0.168
	Male astronauts (n=11)	0.309	0.320
	Female astronauts (n=3)	0.930	0.500
MRS	All astronauts (n=14)	0.123	0.168
	Male astronauts (n=11)	0.236	0.374
	Female astronauts (n=3)	0.466	0.500

*P<0.05 by Spearman's correlation.

Supplementary Table 4. L5-L1 MAT volume gradient in 14 astronauts before and after spaceflight.

	Measures	Preflight -100±43 days	Postflight 41±6 days	Postflight 184±15 days	Postflight 363±25 days
All astronauts (n=14)	PD	9.4 (5.7)	8.2 (6.3)	11.1 (5.8)	11.6 (8.2)
	DIXON	7.0 (2.6)	7.8 (2.3)*	6.9 (1.9)	7.3 (2.4)
	Both techniques	8.19 (4.5)	8.03 (4.7)	9.01 (4.6)	9.48 (6.2)
Male astronauts (n=11)	PD	10.1 (5.7)	8.6 (6.6)	11.3 (6.2)	14.2 (7.4)#
	DIXON	7.0 (2.6)	8.0 (2.4)*	7.2 (1.6)	7.7 (2.4)
	Both techniques	8.55 (4.6)	8.30 (4.9)	9.24 (4.8)	10.91 (6.2)#
Female astronauts (n=3)	PD	5.8 (5.8)	6.9 (6.5)	10.3 (5.1)	2.9 (3.7)#
	DIXON	7.0 (4.0)	7.5 (2.3)	6.1 (2.9)	6.1 (2.4)
	Both techniques	6.37 (4.1)	7.16 (4.4)	8.20 (4.4)	4.49 (3.3)#

Average (SD). *P<0.05 compared to preflight by two-sided Wilcoxon Signed Rank test (n=14) with no adjustment for multiple comparisons. #P<0.05 between male and female astronauts by two-sided Mann-Whitney U test (n=14) with no adjustment for multiple comparisons.

Supplementary Table 5. Whole body lean and fat mass before and after spaceflight.

	Mass (kg)	Preflight 1-3 months	Postflight 3-18 days	Postflight 6-9 months	Postflight 1 year
All astronauts (n=14)	Lean	57.4 (7.9)	59.3 (7.4)	61.2 (6.7)	62.0 (7.6)
	Fat	23.2 (5.2)	21.0 (5.6)	24.4 (5.9)	25.6 (7.5)
Male astronauts (n=11)	Lean	61.7 (7.1)	62.1 (6.2)#	63.8 (5.3)	64.3 (5.8)
	Fat	21.3 (1.6)	19.2 (3.9)	22.2 (4.1)	23.1 (4.9)
Female astronauts (n=3)	Lean	50.9 (3.0)	51.2 (3.3)#	53.1 (0.8)	N/A
	Fat	25.9 (8.7)	26.2 (7.5)	31.1 (6.6)	N/A

Average (SD). #$P<0.05$ between male and female astronauts by two-sided Mann-Whitney U test (n=14) with no adjustment for multiple comparisons.
