# Supplementary material for: Bone marrow adiposity modulation after long duration spaceflight in astronauts
Source: Nat Commun. 2023 Aug 9;14:4799. doi: 10.1038/s41467-023-40572-8 (PMC10412640; doi:10.1038/s41467-023-40572-8)
Supplement: Supplementary file 3 — Description of Additional Supplementary Files [file 41467_2023_40572_MOESM3_ESM.pdf]

Description of Additional Supplementary File Document

Supplementary Data 1. Outcome measures of 14 astronauts preflight, inflight, and postflight.
