# Supplementary material for: Bone marrow adiposity modulation after long duration spaceflight in astronauts
Source: Nat Commun. 2023 Aug 9;14:4799. doi: 10.1038/s41467-023-40572-8 (PMC10412640; doi:10.1038/s41467-023-40572-8)
Supplement: Supplementary file 5 — Reporting Summary [file 41467_2023_40572_MOESM5_ESM.pdf]

nature portfolio

Corresponding author(s): Guy Trudel

Last updated by author(s): Jul 10, 2023



# Reporting Summary

Nature Portfolio wishes to improve the reproducibility of the work that we publish. This form provides structure for consistency and transparency in reporting. For further information on Nature Portfolio policies, see our Editorial Policies and the Editorial Policy Checklist.

## Statistics

For all statistical analyses, confirm that the following items are present in the figure legend, table legend, main text, or Methods section.

| n/a | Confirmed | |
|---|---|---|
| ☐ | ☒ | The exact sample size (*n*) for each experimental group/condition, given as a discrete number and unit of measurement |
| ☐ | ☒ | A statement on whether measurements were taken from distinct samples or whether the same sample was measured repeatedly |
| ☐ | ☒ | The statistical test(s) used AND whether they are one- or two-sided<br>*Only common tests should be described solely by name; describe more complex techniques in the Methods section.* |
| ☐ | ☒ | A description of all covariates tested |
| ☐ | ☒ | A description of any assumptions or corrections, such as tests of normality and adjustment for multiple comparisons |
| ☐ | ☒ | A full description of the statistical parameters including central tendency (e.g. means) or other basic estimates (e.g. regression coefficient) AND variation (e.g. standard deviation) or associated estimates of uncertainty (e.g. confidence intervals) |
| ☐ | ☒ | For null hypothesis testing, the test statistic (e.g. $F$, $t$, $r$) with confidence intervals, effect sizes, degrees of freedom and $P$ value noted<br>*Give P values as exact values whenever suitable.* |
| ☒ | ☐ | For Bayesian analysis, information on the choice of priors and Markov chain Monte Carlo settings |
| ☒ | ☐ | For hierarchical and complex designs, identification of the appropriate level for tests and full reporting of outcomes |
| ☐ | ☒ | Estimates of effect sizes (e.g. Cohen's *d*, Pearson's *r*), indicating how they were calculated |

*Our web collection on statistics for biologists contains articles on many of the points above.*

## Software and code

Policy information about availability of computer code

| | |
|---|---|
| Data collection | MRI was obtained using a 3T scanner (Magnetom Verio, Software version VB19A, Siemens Healthineers, Erlangen, Germany) and (Biograph nMR, Software version VE11, Siemens Healthineers, Erlangen, Germany). |
| Data analysis | MRI vertebral fat fraction data collection was done using in-house segmentation software programs developed in Matlab 2014a. MR spectroscopy was processed using LCModel (6.3-1L; Oakville, Canada). For linewidth and frequency analysis, the MR spectroscopy was processed using the AMARES fitting algorithm from the jMRUI software package (v6.0 beta). Data analysis was done using SPSS Statistics 27.0. |

For manuscripts utilizing custom algorithms or software that are central to the research but not yet described in published literature, software must be made available to editors and reviewers. We strongly encourage code deposition in a community repository (e.g. GitHub). See the Nature Portfolio guidelines for submitting code & software for further information.

## Data

Policy information about availability of data

All manuscripts must include a data availability statement. This statement should provide the following information, where applicable:

- Accession codes, unique identifiers, or web links for publicly available datasets
- A description of any restrictions on data availability
- For clinical datasets or third party data, please ensure that the statement adheres to our policy

Aggregated data to understand and access the conclusions of this research are available in the figures and supplementary tables. Individual astronaut source data

have been deposited in NASA's Life Sciences Data Archives. Investigators can request access to the astronaut data at https://lsda.jsc.nasa.gov/. Both de-identified and possibly attributable (identifiable) astronaut data may be available for internal and external-to-NASA peer-reviewed research studies following NASA IRB approval. The length of time required to complete a data request is based on the complexity of the request and volume of the data requested.

## Research involving human participants, their data, or biological material

Policy information about studies with human participants or human data. See also policy information about sex, gender (identity/presentation), and sexual orientation and race, ethnicity and racism.

| | |
|---|---|
| Reporting on sex and gender | Sex-based analyses were performed on the astronaut data. Sex was determined based on self-reporting. Sex was considered in the study design but a sex-balanced study design was limited due to the availability of astronauts at the time of informed consent briefing. |
| Reporting on race, ethnicity, or other socially relevant groupings | Race, ethnicity, or other socially relevant groupings were not reported. |
| Population characteristics | All astronauts participated in a planned 6 month excursion on board the International Space Station. 14 astronauts were recruited (11 men and 3 women; 45±7 years). |
| Recruitment | Between 2015 and 2020, astronauts were consecutively interviewed approximately 1 year ahead of their ISS missions. 20 astronauts were presented with the MARROW protocol. 15 astronauts agreed to participate and signed a written consent form to participate in the study. No participation compensation was provided. 1 astronaut was not provided with a mission during the duration of the MARROW study. There are fewer women than men in the astronaut corps. Our recruitment was representative with more male astronauts recruited. Each individual astronaut could have elected to participate in a complement of different experiments, some of which might have included additional venipunctures, causing blood and iron losses. |
| Ethics oversight | Ottawa Hospital Science Network Research Ethics Board (OHSN-REB) Protocol # 2009646-01H, Johnson Space Center Institutional Review Board (JSC-IRB) Pro 1283, Human Research Multilateral Review Board (HRMRB) Pro 1283, European Space Agency Medical Board (ESA MB) MARROW study, and Japanese Aerospace Exploration Agency (JAXA MB) JX-IRBA-20-04. |

Note that full information on the approval of the study protocol must also be provided in the manuscript.

# Field-specific reporting

Please select the one below that is the best fit for your research. If you are not sure, read the appropriate sections before making your selection.

☒ Life sciences ☐ Behavioural & social sciences ☐ Ecological, evolutionary & environmental sciences

For a reference copy of the document with all sections, see nature.com/documents/nr-reporting-summary-flat.pdf

# Life sciences study design

All studies must disclose on these points even when the disclosure is negative.

| | |
|---|---|
| Sample size | 20 astronauts were presented with the MARROW protocol. 15 astronauts agreed to participant. 1 astronaut was not provided with a mission during the duration of the MARROW study. 11 male and 3 female astronauts completed the trial.<br><br>The minimum required number of subjects needed to meet our primary scientific objectives is nine (9). This estimate is based on a previous bedrest study where the change in fat fraction was 3.6±1.2% SEM at the end of 60 days of bed rest. With 9 subjects we will have 80% power to detect an absolute change of 3.4% in fat fraction. This is a plausible expected change after a 6 months sojourn in microgravity. However, exercise routines could blunt this effect and we may need a higher sample size to detect a significant change. We will target 9+1 for a total of 10 subjects. With less than 8 subjects we will likely be unable to obtain statistical significance for the main outcome measure of bone marrow fat accumulation. |
| Data exclusions | No data were excluded. |
| Replication | The fat fraction at each vertebra level was measured 3 times across 3 interpedicular slices. |
| Randomization | Randomization was not applicable as this is an observational study. Astronauts recruited to the MARROW protocol performed serial measures at specified time points preflight, onboard the ISS, and postflight up to 1 year after their space missions. |
| Blinding | The investigators involved in this study were not blinded. Individuals involved in the data collection and analysis were blinded. Each participant was assigned a random study number. Aggregate data are presented into of specific age, date, or flight duration to prevent attributability. |

# Reporting for specific materials, systems and methods

We require information from authors about some types of materials, experimental systems and methods used in many studies. Here, indicate whether each material, system or method listed is relevant to your study. If you are not sure if a list item applies to your research, read the appropriate section before selecting a response.

## Materials & experimental systems

| n/a | Involved in the study |
| --- | --- |
| ☒ | ☐ Antibodies |
| ☒ | ☐ Eukaryotic cell lines |
| ☒ | ☐ Palaeontology and archaeology |
| ☒ | ☐ Animals and other organisms |
| ☐ | ☒ Clinical data |
| ☒ | ☐ Dual use research of concern |
| ☒ | ☐ Plants |

## Methods

| n/a | Involved in the study |
| --- | --- |
| ☒ | ☐ ChIP-seq |
| ☒ | ☐ Flow cytometry |
| ☒ | ☐ MRI-based neuroimaging |

# Clinical data

Policy information about clinical studies

All manuscripts should comply with the ICMJE guidelines for publication of clinical research and a completed CONSORT checklist must be included with all submissions.

| | |
| --- | --- |
| Clinical trial registration | The study was registered at NASA ethics # Pro1283 at https://lsda.jsc.nasa.gov/Experiment/exper/13399 |
| Study protocol | The protocol can be found at https://lsda.jsc.nasa.gov/Experiment/exper/13399 |
| Data collection | Preflight baseline data collection (BDC) is planned to occur between Launch – 90 days (L-90) and L-60. MRIs will take place on recovery days R+30 ±7, R+180 ±30, and R+365 ±30. |
| Outcomes | To measure fat accumulation in the bone marrow on astronauts pre- and post- stay aboard the International Space Station (ISS) using non-invasive magnetic resonance imaging (MRI). |
